# Exploring the potential bioactive compounds group and mechanism of Ci Bai Capsule in treating leukopenia: a combined approach of network pharmacology and transcriptome evidences

**DOI:** 10.1186/s13020-025-01197-9

**Published:** 2025-09-01

**Authors:** Dingfan Zhang, Congshu Huang, Lei Zhou, Boyang Wang, Wei Zhou, Tiantian Xia, Pan Shen, Shao Li, Yue Gao

**Affiliations:** 1 Institute for TCM-X, MOE Key Laboratory of Bioinformatics/Bioinformatics Division, BNRIST, Department of Automation, Tsinghua University, Beijing, 100084 China; 2 Beijing Institute of Radiation Medicine, Beijing, 100850 China; 3 State Key Laboratory of Kidney Diseases, Chinese PLA General Hospital, Beijing, 100853 China; 4 College of Traditional Chinese Medicine, Henan University of Chinese Medicine, Zhengzhou, 455046 China; 5 Medical College, Qinghai University, Xining, 810016 China

**Keywords:** Ci Bai Capsule, Network target, Leukopenia, Transcriptomics, RNA-seq analysis

## Abstract

**Background:**

Radiation-induced leukopenia caused by low-dose exposure is frequently associated with Traditional Chinese Medicine (TCM) syndromes like “blood deficiency” and “fatigue syndrome”. Ci Bai Capsule (CB) has been reported to enhance white blood cell levels; however, its mechanisms and bioactive compounds remain unclear.

**Aim:**

This study aimed to identify the bioactive compounds group of CB and elucidate its potential mechanisms in radiation-induced leukopenia.

**Methods:**

Syndrome-related data were gathered from SYMMAP and CTD database. CB’s target profile is predicted by DrugCIPHER. Network pharmacology approaches were employed to identify active compounds and related pathways. Experimental validation was conducted through flow cytometry, RNA-sequencing both ex vivo and in vivo models. RT-qPCR and Western blot were performed for quantitative validation of key targets.

**Results:**

A total of 22 pathways related to cellular processes, immune responses, and signal transduction were identified. Five key bioactive compounds (kaempferol-3-glucorhamnoside, syringin, schisandrin, 3-hydroxytyrosol 3-*O*-glucoside and salidroside) were found to significantly modulate syndrome-related pathways. Optimal dosing of this compound combination enhanced leukocyte counts and splenic immune cell proliferation in irradiated mice. Transcriptomic analysis revealed that the compounds exert regulatory effects on PP1A, RB, CDK4/6, CDK2, and CDK1, thereby modulating downstream immune and hematopoietic markers such as MNDA, BST2, and HSPA1A.

**Conclusion:**

Our findings suggest that CB mitigates radiation-induced leukopenia by enhancing immune and hematopoietic recovery, offering a promising therapeutic approach for managing radiation-related hematological disorders.

**Supplementary Information:**

The online version contains supplementary material available at 10.1186/s13020-025-01197-9.

## Introduction

In clinical practice, fractionated low-dose radiation (0.5–2 Gy) has garnered significant attention due to its capacity to enhance the efficacy of tumor checkpoint blockade immunotherapy, thereby delaying or inhibiting the progression of primary and metastatic cancers [58]. Radiation doses below 2 Gy are generally defined as low-dose radiation in clinical radiotherapy, representing the most frequently applied dose range in therapeutic protocols [31]. However, radiation-induced leukopenia occurring in over 20% of radiotherapy patients [70] manifests as critically low white blood cell (WBC) counts due to radiation exposure [51, 75], which compromises the immune system and increases the risk of infection, treatment interruption, and poor clinical outcomes [9, 10, 55]. This disruption arises from dual radiation effects: directly hematopoietic stem cell damage and microenvironmental impairment in spleen and bone marrow, which governs WBC storage, proliferation, and activation [53, 69]. Thus, developing protective measures is crucial. However, current commonly used therapeutic options remain limited in variety and demonstrate suboptimal efficacy and side effects [6, 10, 17]. Current clinical strategies to manage radiation-induced leukopenia primarily include dose adjustment of radiation, administration of hematopoietic growth factors [6, 18] such as granulocyte colony-stimulating factor (G-CSF), and supportive care. While G-CSF effectively promotes neutrophil recovery, its use is often limited by high costs, short duration of action, and potential adverse effects, including bone pain and splenic rupture [17]. Moreover, these interventions mainly target symptom relief without addressing the underlying hematopoietic dysfunction. In recent years, traditional Chinese medicine (TCM) has been increasingly explored as a complementary approach [66], aiming to enhance hematopoiesis, modulate immune responses, and improve patient quality of life.

Ci Bai Capsule (CB), a traditional Chinese medicine (TCM) formula derived from the ancient prescriptions “Si Jun Zi Tang” [35] and “Wu Zi Yan Zong Pill” [79], emerges as a promising alternative treatment due to its immunomodulatory effects. Clinical evidence has confirmed the CB’s efficacy in treating leukocyte reduction caused by radiation and chemotherapy [26]. The core herbs in CB, including *Scleromitrion diffusum* (BHSSC), *Eleutherococcus senticosus* (CWJ) and *Poria* (FL), work synergistically to tonify Qi [34], nourish blood [41], and enhance hematopoiesis [56]. Consequently, CB is deemed capable of strengthening the spleen, restoring immune balance, and boosting the body’s natural defenses, thereby demonstrating particular effectiveness in managing leukopenia. However, the unclear pharmacological mechanisms hinder its clinical application and international recognition.

Network pharmacology offers a systematic and holistic approach to address the complexity of multicomponent herbal medicines by integrating molecular interaction data with pharmacological insights [60, 77, 78]. The “Network Target” theory enables the identification of bioactive compounds and their corresponding therapeutic mechanisms, providing a robust framework for exploring the efficacy of traditional formulas [39, 40, 77]. This approach enables the resolution of the therapeutic potential of CB in treating radiation-induced leukopenia by combining network pharmacology with TCM syndrome analysis, aiming to uncover the active compound combinations and underlying mechanisms that contribute to its efficacy.

In this study, we systematically investigated the therapeutic mechanisms of CB against radiation-induced leukopenia through network pharmacology analysis. By analyzing CB’s multi-component interactions, we identified potential regulatory networks within radiation-affected immune and hematopoietic systems. A pharmacological screening method incorporating TCM syndrome characteristics of leukopenia was applied to prioritize key bioactive compounds. Experimental validation further revealed CB’s dual mechanisms of leukogenic action. These findings clarify CB’s therapeutic basis while providing a methodological approach for evaluating complex herbal formulations, contributing to the development of mechanism-informed TCM therapies for radiation injuries.

## Methods and materials

### Data collection

Data of syndromes were gathered from the SYMMAP [73] database, identifying OMIM standard entries related to TCM syndromes associated with leukopenia. For example, within the framework of TCM theory, the TCM syndrome “weakened function of the spleen and kidneys” in SYMMAP is related with phenotypes like Asthenia, Diarrhea, vision_low and Mental Deficiency. Each phenotype has its own standard Mesh term. Furthermore, based on the comparative toxicogenomics database (CTD) [19], genes linked to each Mesh term were identified and designated as genes associated with the TCM syndrome.

### Target prediction and validation

The DrugCIPHER algorithm [80], which is based on chemical similarity and network-driven drug target prediction, was employed to predict the genome-wide targets of each chemical constituent in CB. The top 100 predicted targets for each compound were considered as the compound’s target profile. To explore the co-occurrence relationships between each chemical component and its predicted targets, abstracts from the PubMed database and drug-target data from the PubChem database were utilized. These co-occurrences were systematically organized to assess the extent of literature coverage for the predicted targets. The existing literature coverage was then leveraged to evaluate the predictive accuracy of each compound’s target identification. Accuracy was calculated according to the following formula:

1 $$ {\text{Accuracy}} = { }\left( {\frac{Number\;of\;intersections\;between\;predicted\;targets\;and\;reported\;biomolecules}{{Total\;number\;predicted\;targets}}} \right) \times 100\% $$

### Network target analysis and multilayer network construction

Statistical analyses were performed under the hypothesis that targets present in a higher number of major component compounds within formulations are more likely to represent key therapeutic targets. This approach facilitated the construction of a holistic target profile for the formula [44], allowing for the identification of targets that may play a pivotal role in its pharmacological effects. The results provided an integrated overview of the target landscape associated with the TCM formulation:

2 $$ p_{r} \left( {K = k} \right) = \mathop \sum \limits_{{A \in F_{k} }} \mathop \prod \limits_{i \in A} p_{i} \mathop \prod \limits_{{j \in A^{c} }} \left( {1 - p_{j} } \right) $$

where, ${p}_{r}\left(K=k\right)$ is the probability that a target protein occurs in the target profiles of k ingredients, ${F}_{k}$ is the set of all subsets of k ingredients, A is one particular subset of k ingredients, and ${A}^{c}$ is the complement of A. ${p}_{i}$ and ${p}_{j}$ are the probabilities of a target protein being contained in the target profiles of an ingredient.

The identified holistic targets were subsequently subjected to KEGG pathway [28] and gene ontology (GO) term [4] enrichment analyses to identify key biological pathways and processes potentially involved in the treatment of leukopenia by CB. Integrating these results with RNA-seq data, relevant pathways and biological processes associated with leukopenia were selected from the enriched targets of CB. These pathways and processes were classified into three distinct biological modules according to their functional roles.

To further elucidate the molecular interactions, a protein–protein interaction (PPI) network was constructed using the STRING database [47], focusing on proteins enriched in each module’s pathways. The resulting network was visualized using Cytoscape (v3.9.1) software [64]. Based on TCM principles of compatibility, the herbal components of CB were categorized into three roles: sovereign (JUN), minister (CHEN), assistant (ZUO/SHI). The holistic targets associated with each category of herb were calculated and linked to the corresponding target columns. Furthermore, the association of these targets with specific pathways within the biological modules highlighted the enrichment of these targets in particular signaling pathways relevant to the treatment of leukopenia.

### Selection of potential bioactive compounds group (BCG)

Compounds with significant therapeutic effects on the TCM syndromes of leukopenia were identified and selected to exemplify the therapeutic actions of CB. Based on previously collected “syndrome-genes” data, four distinct gene datasets corresponding to TCM syndromes were established, including febrile disease (FD), weakened spleen and kidneys (WSK), insufficient blood (IB), and chronic deficiency or fatigue syndrome (CDFS). The enrichment of the compounds’ target profiles within each dataset was evaluated using hypergeometric distribution principles, as outlined in Eq. (3). A significant result (*P* value < 0.05) indicated a potential therapeutic effect of the compound on the respective syndrome.

Furthermore, compounds that showed significant enrichment in any of the datasets were selected for further analysis based on their roles as sovereign ingredients and their inclusion as quality control compounds in the Chinese Pharmacopoeia. These criteria ensured the relevance of the selected compounds to the clinical application and quality standards of CB.

3 $$ p{ - }value = P\left[ {i \ge k} \right] = \mathop \sum \limits_{i = k}^{{min\left( {K,n} \right)}} \frac{{\left( {\begin{array}{*{20}c} K \\ i \\ \end{array} } \right)\left( {\begin{array}{*{20}c} {N - K} \\ {n - i} \\ \end{array} } \right)}}{{\left( {\begin{array}{*{20}c} N \\ n \\ \end{array} } \right)}} $$

where N is the total number of background genes, K is the number of genes associated with a given pathway or functional category, n is the number of genes in the query set, k is the number of genes shared between the query set and the functional category.

### Animal irradiation and treatment

Eighty male C57BL/6 mice of 8 weeks weighing 20 ± 2 g were purchased from GemPharmatech Co. Itd (Nanjing, China). All animal experiments conducted in this study received approval from the Animal Care and Use Committee of the Academy's Animal Center. The mice were maintained under a controlled environment with a 12-h light/dark cycle at room temperature (25 ± 2 °C) and humidity (55 ± 5%), with unrestricted access to food and water. The ^60^Co gamma irradiation source used in the experiment was the Beijing Institute of Radiation Medicine. The mice were housed in a special case behind a shielded lead plate and irradiated with ^60^Co gamma irradiation. A single irradiation dose rate of 2.98 cGy/min, 4m away from the radiation source, irradiation dose of 0.1 Gy, at the same position for 5 consecutive days. The mice were divided into normal control group, irradiation group and irradiation administration group. The control group underwent no treatment, the irradiation administration group was given drug gavage immediately after the completion of irradiation for 5 consecutive days, and the irradiation group was given normal saline gavage.

### Uniform design of experimental scheme

Using U_6_(6^5^) uniform design table, we selected Syringin, Salidroside, Kaempferol, Schisandrol A, and Cimidahurinine as the examined factors. Each factor was assigned 6 levels, and the dosage ranges for Syringin, Salidroside, Kaempferol, Schisandrol A, and Cimidahurinine were determined based on literature [3, 42, 43] to be 10–60 mg kg^−1^, 25–85 mg kg^−1^, 5–40 mg kg^−1^, 5–100 mg kg^−1^, 1–41 mg kg^−1^, respectively. Using the uniform design table (Table 1), we designed a dosage combination experiment plan.

**Table 1:** Uniformly designed and screened the experimental prescription administration scheme

Group	Syringin mg kg^−1^	Salidroside mg kg^−1^	Kaempferol mg kg^−1^	Schisandrol A mg kg^−1^	Cimidahurinine mg kg^−1^
Uniform 1 (A)	10	25	19	62	41
Uniform 2 (B)	20	55	40	5	33
Uniform 3 (C)	30	85	12	81	25
Uniform 4 (D)	40	10	33	24	17
Uniform 5 (E)	50	40	5	100	9
Uniform 6 (Y)	60	70	26	43	1

### Mouse blood cell level detection

The mice were anesthetized with pentobarbital sodium, and after the left eye was removed and blood was collected, it was treated with anticoagulant and analyzed using an automatic hematocrit analyzer.

### Flow cytometric analysis

On the sixth day after irradiation, the mice were euthanized. The thymus and spleen were collected separately and ground to collect cells. The collected cells were washed once and then re-suspended in 100 μL PBS containing 2% fetal bovine serum (FBS). For staining of lymphocytes in the thymus and spleen, the cells were incubated with biotin-conjugated antibodies against CD4 and CD8 for 30 min, followed by incubation with streptavidin-conjugated secondary antibodies and CD45 for 30 min after one wash. The cells were then washed once and re-suspended in PBS containing 0.1% bovine serum albumin. Five minutes before analyzing the cells on the Aria III (BD Biosciences), 5 μL of 7-AAD viability staining solution was used.

### Quantitative real-time PCR

Quantitative real-time PCR was performed as previously described [5]. Total RNA was extracted from mouse spleens using TRIzol reagent, followed by purification and concentration measurement. cDNA was synthesized from RNA samples using a one-step kit that integrates genomic DNA removal and reverse transcription. qPCR amplification was conducted on a real-time PCR system with SYBR Green fluorescence detection. The thermal cycling protocol included an initial denaturation step at 94 °C for 30 s, followed by 40 cycles of denaturation at 94 °C for 5 s, annealing at 60 °C for 15 s, and extension at 72 °C for 10 s. All primer designs were performed on the National Center for Biotechnology Information Search Database (NCBI) website and synthesized by Sangon Bioengineering Co. Ltd. Primer sequences are shown in Supplementary File.

### Western blot assay

Western blot analysis was conducted as previous reported [27]. Mouse spleens were lysed in ice-cold RIPA buffer with protease inhibitors after treatment. Proteins were quantified via BCA assay. Samples (20–40 µg) were resolved by 10% SDS-PAGE and transferred to PVDF membranes. Membranes were blocked with 5% milk in TBST for 2 h, then incubated overnight at 4 °C with primary antibodies. After TBST washes, HRP-conjugated secondary antibodies (anti-rabbit/mouse IgG) were applied for 1 h. Bands were visualized using ECL substrate and imaged on ImageQuant LAS 500.

### RNA-seq analysis

Total RNA was used for RNA sample preparation, with mRNA isolated via poly-T oligo-attached magnetic beads. Fragmentation was performed under elevated temperature with divalent cations in the First Strand Synthesis Reaction Buffer. First strand cDNA synthesis utilized random hexamer primers and M-MuLV Reverse Transcriptase, followed by second strand synthesis with DNA Polymerase I and RNase H. Overhangs were converted to blunt ends, and after adenylation of the 3ʹ ends, adaptors with a hairpin loop structure were ligated. The cDNA fragments were size-selected to a length range of 370–420 bp using the AMPure XP system, followed by PCR amplification with Phusion High-Fidelity DNA polymerase and universal primers. PCR products were purified and library quantification was carried out using Qubit. The effective library concentration was confirmed by qRT-PCR to ensure quality. Qualified libraries were pooled and sequenced based on the required concentration and data output.

For bioinformatics analysis, raw sequencing data were processed using fastp (v0.23.1) to assess quality. The sequence data, obtained in FASTQ format, were examined for adapter contamination, low-quality bases, and uncertain nucleotides. Paired reads were discarded if any of the following criteria were met [74]: (1) either read in a pair contained more than 10% ambiguous nucleotides (N); (2) either read in a pair contained more than 50% of low-quality bases (Q ≤ 5); or (3) either read in a pair contained adapter sequences. These quality control steps ensured that only reliable data were used for downstream analysis.

RNA-seq analysis was conducted based on R package DESeq2 (v1.30.0) and clusterProfler (v4.9.0). Gene set enrichment analysis (GSEA) was used to explore the enrichment of KEGG pathways and biological processes within differential expression results. Genes with |log_2_FoldChange|> 1 and adjusted *P* value < 0.05), as differentially expressed genes (DEGs), were kept for further analysis. Enrichment analysis was used to assess the enrichment levels of target genes in KEGG pathways and biological processes. Immune cell fractions were inferred by CIBERSORT [52] into 22 sub classes with default parameters.

### Statistical analysis

This study utilized Poisson binomial, Pearson correlation, and hypergeometric distribution statistical models. Fisher’s exact test, with Benjamini–Hochberg correction for multiple testing, was the primary method applied for statistical analysis.

## Results

### Integrated compound identification and target validation reveals CB’s multi-component synergy

**Fig. 1:**
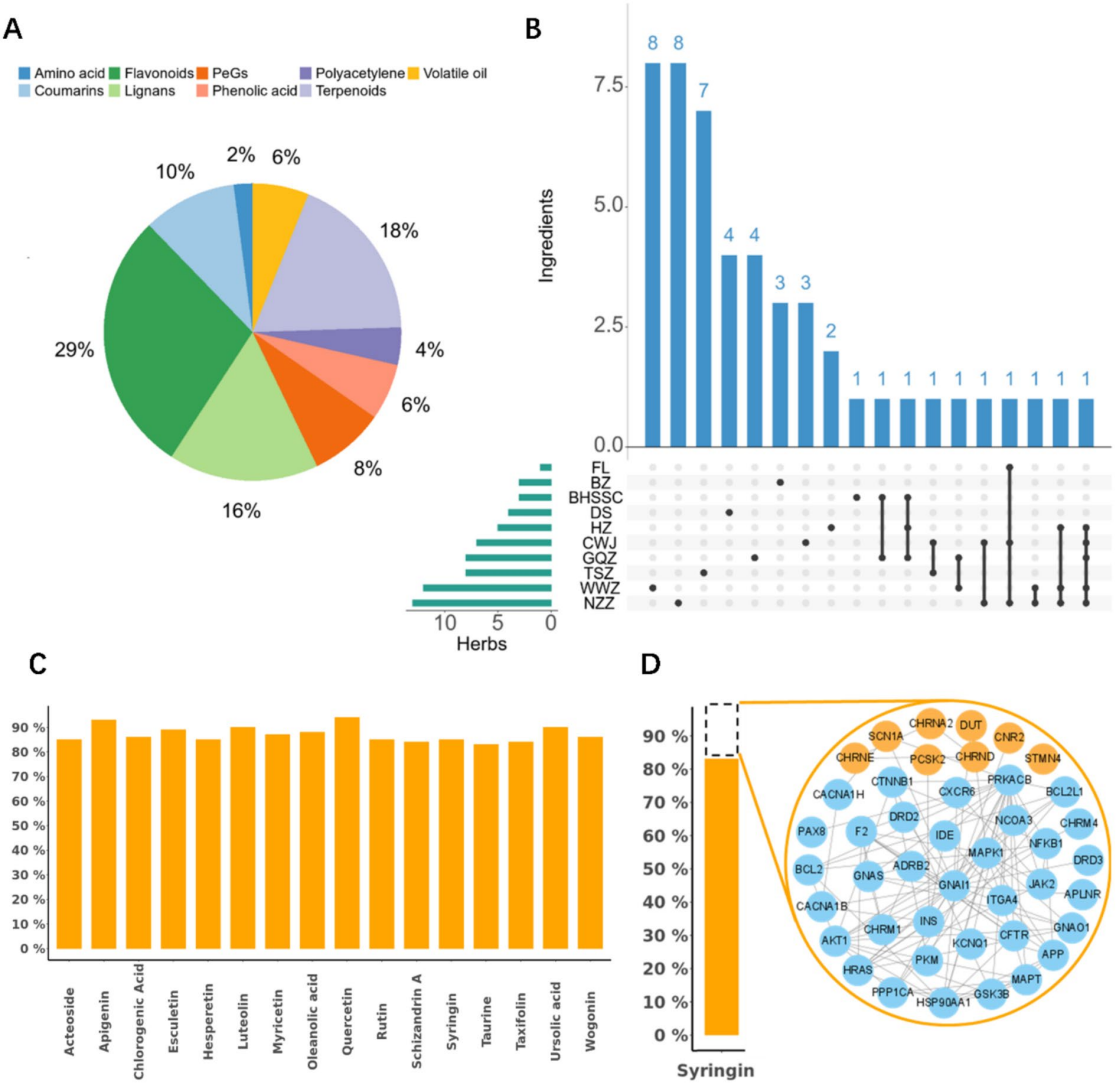
Compound identification, targets prediction and literature validation of predicted targets of compounds in CB. **A** Pie chart of the chemical classes to which the 49 identified chemical components of CB belonged. **B** UpSet plot of the herb classes to which the 49 identified chemical components belonged. **C** Target prediction and validation of compounds in CB, displaying literature coverage rate (85–97%) of the main chemical components. **D** The reported and unreported predicted targets of syringin in the same biomolecular network

Previous study of CB revealed a total of 49 chemical components, classified into nine distinct groups according to their chemical structures (see Supplementary File). The compound groups ordered by descending proportion were as follows: flavonoids (14/49, 28.6%), terpenoids (9/49, 18.4%), lignans (8/49, 16.3%), coumarins (5/49, 10.2%), phenylethanoid glycosides (aka. PeGs) (4/49, 8.1%), phenolic acid (3/49, 6.1%), volatile oil (3/49, 6.1%), polyacetylene (2/49, 4.1%), and amino acid (1/49, 2.0%) (Fig. 1A). To elucidate the distribution of compounds across various herbs, we employed an UpSet plot (Fig. 1B) to visualize the number of compounds identified within each herb and their common presence across different herbs. The results demonstrated that the majority of these compounds are derived from *Scleromitrion diffusum* (BHSSC), *Eleutherococcus senticosus* (CWJ), *Poria* (FL), *Atractylodes macrocephala* Koidz (BZ), *Codonopsis radix* (DS), *Reynoutria japonica* (HZ), *Lycium chinense* (GQZ), *Cuscuta* (TSZ), *Schisandra chinensis* (WWZ), *Ligustri lucidi* fructus (NZZ). Notably, certain chemical components, such as Quercetin, are found across multiple herbs exemplifying shared components. This compound, for instance, is present in five herbs used in the CB highlighting its widespread relevance in these herbs.

Based on the components in CB, the DrugCIPHER algorithm was employed to predict the target profile of each identified compound. A comparative analysis between the predicted target profile and those documented in the literature revealed an overlap of over 80%, providing strong validation for the algorithm’s predictive accuracy (Fig. 1C). Specifically, for syringin, 82% of the predicted targets were either directly supported or indirectly connected through documented protein interactions and signaling pathways. The remaining 18% were linked within a complex biomolecular network, further corroborating the reliability and robustness of the DrugCIPHER predictions (Fig. 1D).

### Transcriptomic analysis elucidates immune dysfunction in low-dose radiation-induced leukopenia

**Fig. 2:**
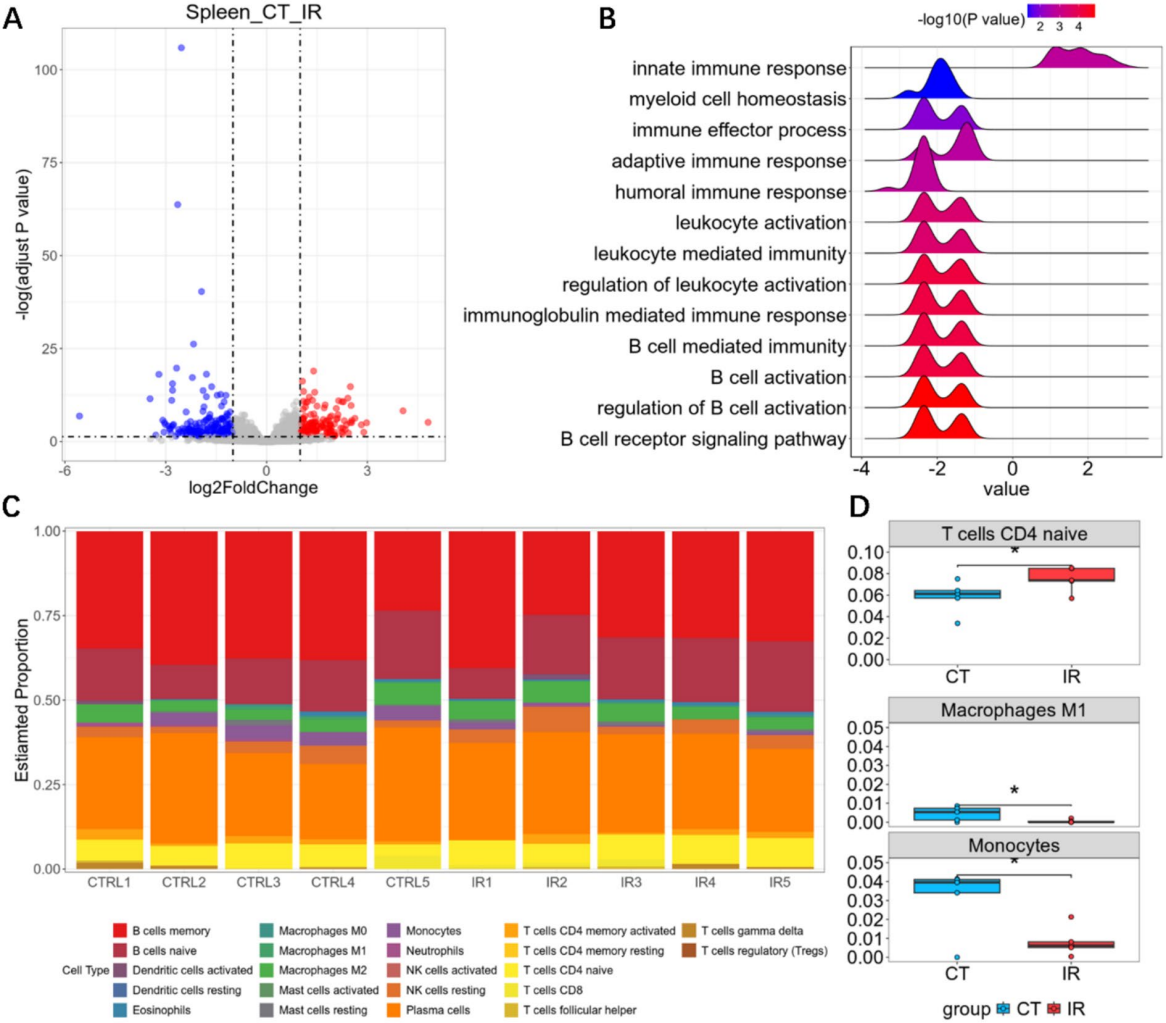
RNA-seq analysis of low-dose radiation-induced leukopenia. **A** Volcano plots for differential expressed genes before and after radiation in spleen. **B** Enrichment result of the significantly changed biological processes before and after radiation in spleen. **C** Bar plot showing the proportions of immune cells in different samples before and after radiation in spleen. **D** Inferred proportions of naïve T cells, macrophages and monocytes before and after radiation in spleen

The spleen, as the largest secondary lymphoid organ playing a central role in immune regulation [37] and hematopoiesis [63], suffers radiation-induced microenvironmental damage that critically contributes to leukopenia development by disrupting both immune regulation and blood cell regeneration [1, 24, 71]. To investigate the mechanisms of low-dose radiation-induced leukopenia, we obtained and analyzed the RNA-seq data of mice spleen before and after radiation exposure. Differential expression analysis identified 155 genes that were significantly downregulated and 108 genes that were upregulated in the irradiated group compared to controls (Fig. 2A). GSEA further suggested that radiation exposure disrupts key pathways involved in immune regulation and hematopoiesis (Fig. 2B). Notably, immune-related pathways, such as B cell-mediated immunity and B cell activation, were significantly enriched, emphasizing the impact of low-dose radiation on splenic immune function.

To further investigate cellular changes underlying these transcriptomic alterations, immune cell deconvolution of the RNA-seq data was performed to assess proportional shifts among 22 immune cell subtypes within the spleen (Fig. 2C). Radiation exposure resulted in significant alterations in the proportions of specific immune cell populations, including a marked reduction in monocytes, macrophages, and naïve CD4^+^ T cells (Fig. 2D). These findings suggest that low-dose radiation disrupts immune cell composition and homeostasis in the spleen, impairing its immune regulatory functions. Collectively, these results provide mechanistic insights into the development of leukopenia and the associated immune dysfunction following radiation exposure [16, 54, 59].

### Network target analysis elucidates CB’s multi-pathway mechanism governing regulation of leukopenia through herb-target-function triad

**Fig. 3:**
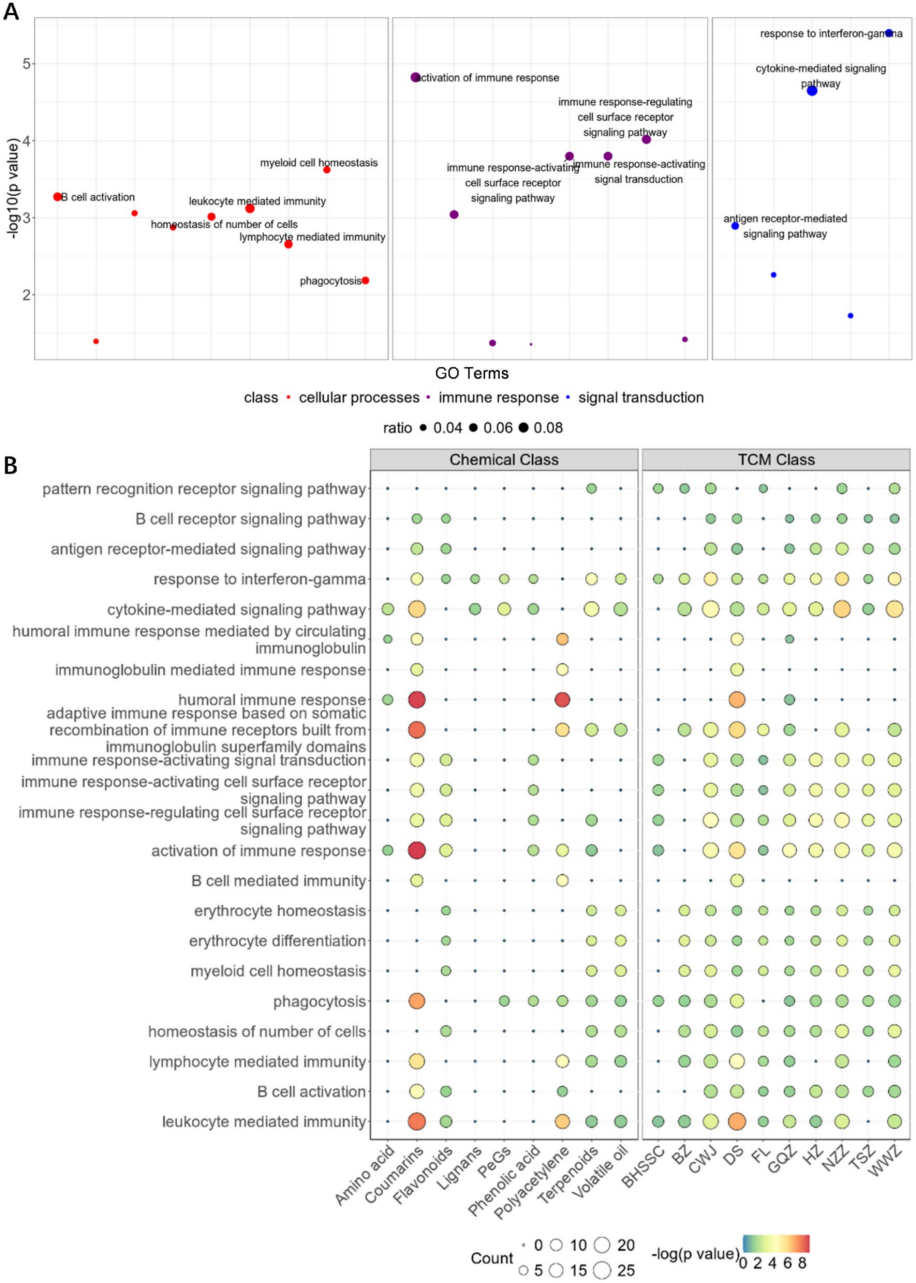
Network target analysis of CB in modules of leukopenia. **A** Enrichment result of pathways and biological processes in different modules of CB in the treatment of leukopenia. **B** Enrichment result of pathways and biological processes in different modules of chemical and herb classes in the treatment of leukopenia

Integrating the GSEA results with enrichment analysis of CB’s holistic target, we identified that CB modulates leukopenia through 22 potential pathways encompassed within three classes: cellular process, immune response, and signal transduction (Fig. 3A). Within cellular process, CB impacts the cellular activities of B cells, leukocytes, and myeloid cells, notably influencing myeloid cell homeostasis to maintain bone marrow cell stability and promote their growth and differentiation, thereby enhancing leukocyte production. In terms of immune response, pathways such as the immune response-activating cell surface receptor signaling pathway regulate immune responses, augmenting the body’s immune function and promoting the activation and proliferation of immune cells. The holistic targets were also enriched in the leukocyte-mediated immunity pathway, indicating the potential of the formulation to enhance the functionality of immune cells such as neutrophils and monocytes, thereby improving the efficacy of the immune system. For signal transduction, CB modulates immune responses and validation processes through pathways such as the interferon-γ mediated signaling pathway and cytokine-mediated signaling pathways. These results indicated that CB demonstrates a multifaceted approach in modulating leukopenia through various pathways, highlighting its potential as a comprehensive therapeutic agent for this condition.

**Fig. 4:**
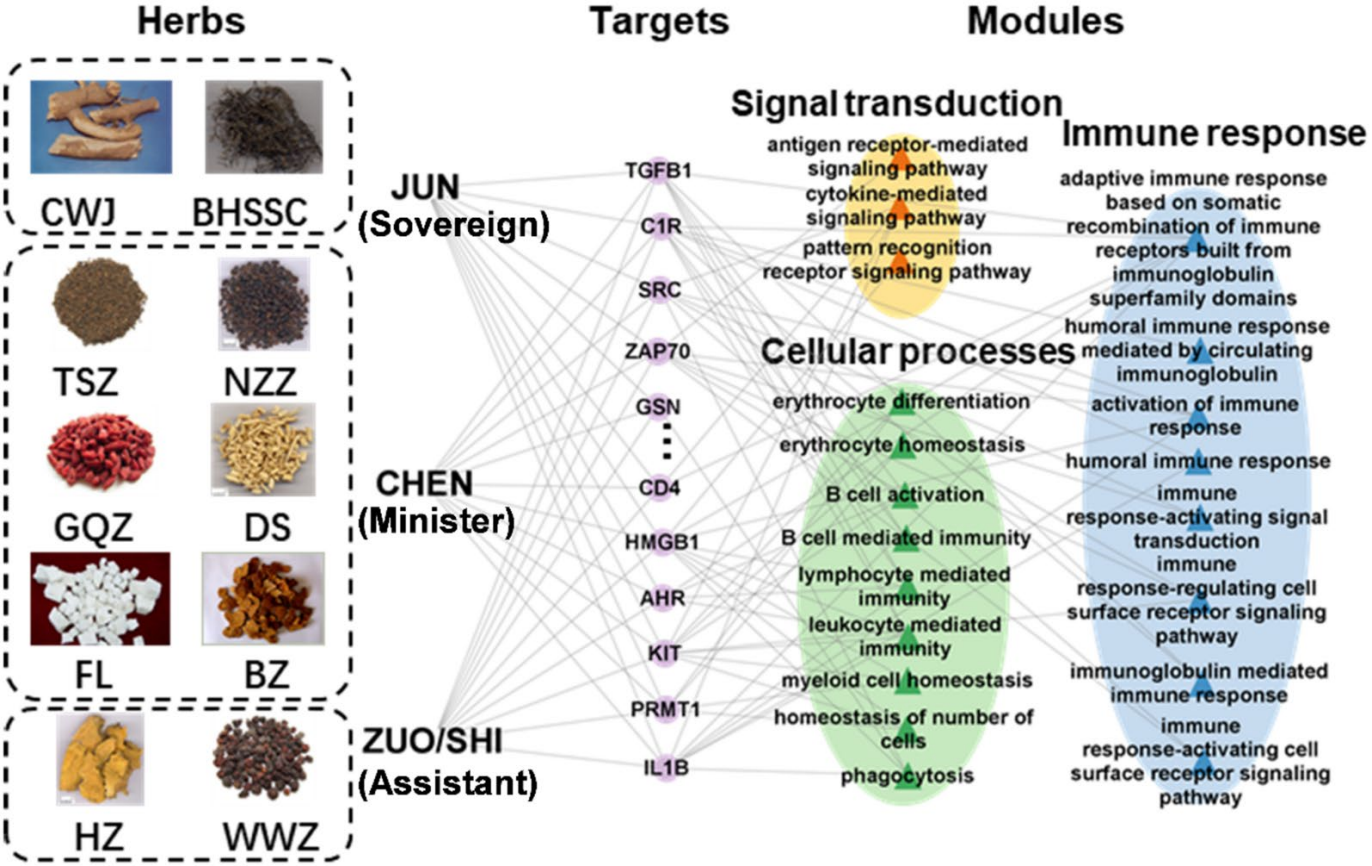
Multi-layer network representing the potential target of CB in the treatment of leukopenia. The left boxes listed the herbs in CB classed by JUN, CHEN, ZUO/SHI. The middle dots showed the potential targets of these herbs, and the right triangles indicated functions of these targets

Further analysis of the compounds in CB according to compound types and their respective herbal sources revealed significant intervention roles of coumarin compounds in signal transduction and immune response modules, while terpenes, flavonoids, and volatile oil compounds showed significant activity in the cellular process module (Fig. 3B). The herbs in CB can be grouped into four categories based on their roles in disease intervention and their relationships with other herbs in the formula: sovereign (JUN), minister (CHEN) and assistant (ZUO/SHI). In CB, the sovereign herbs are BHSSC and CWJ, while the minister herbs include FL, BZ, DS, GQZ, TSZ, and NZZ, with HZ and WWZ classified as assistant herbs. Enrichment analysis indicated that DS, as a minister herb, demonstrated significant effects across all three modules, thereby assisting the sovereign herbs in enhancing the therapeutic efficacy of CB. Notably, the sovereign herb CWJ, the minister herb NZZ, and the assistant and guide herb WWZ exhibited pronounced activity on the cytokine-mediated signaling pathway (Fig. 3B). These three herbs, categorized in TCM as Qi tonics, are known for their ability to promote immune function [23, 30], as well as their antioxidant and anti-aging properties [49]. Building upon the multilayer module network constructed, we identified key targets of CB herbs in the treatment of leukopenia, including TGFB1, C1R, CD4, and HMGB1, which regulate leukopenia through signal transduction, cellular process, and immune response (Fig. 4).

### TCM syndromes-guided discovery indicated the multi-target properties of potential bioactive compounds group derived from CB

**Fig. 5:**
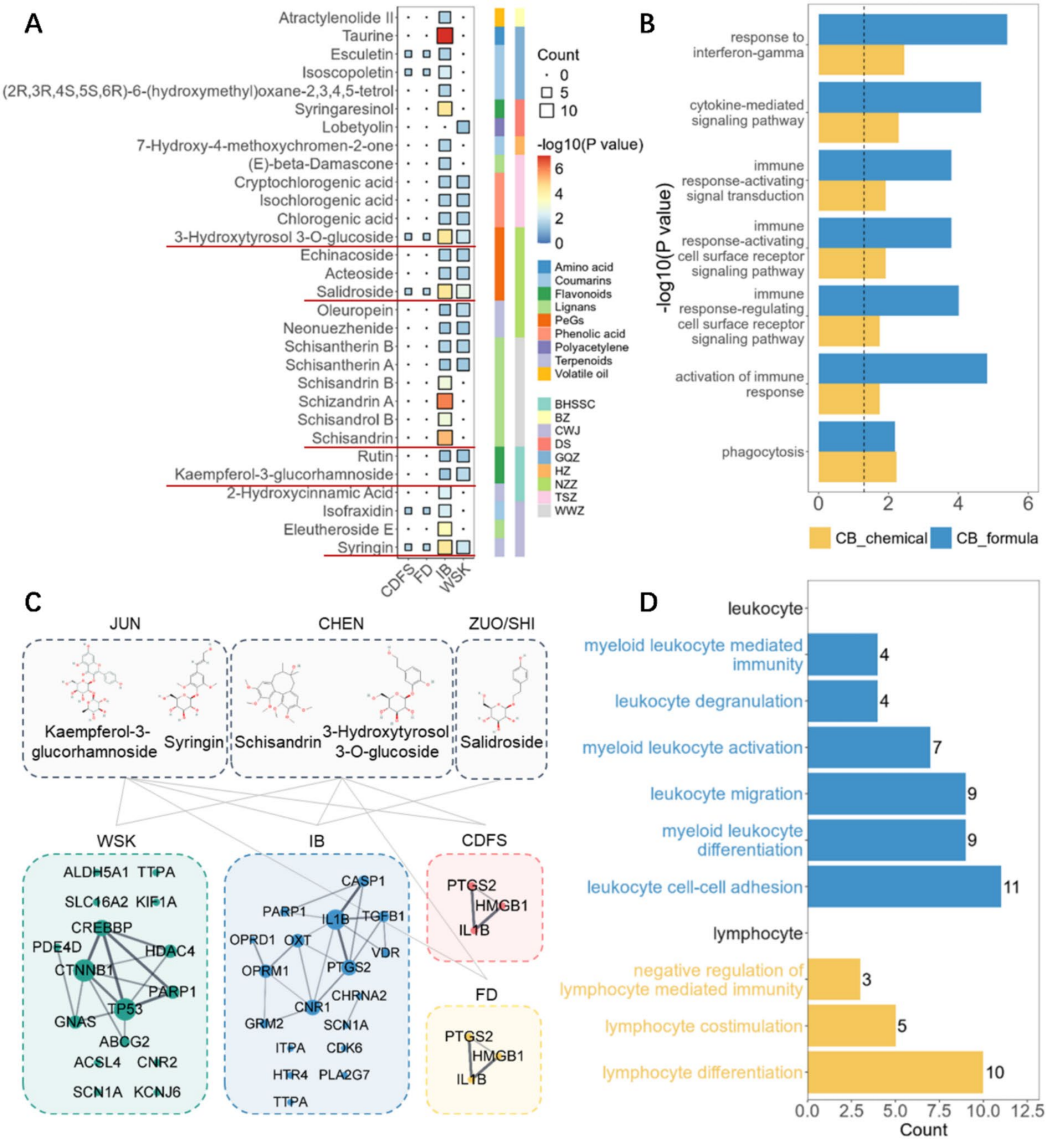
Identification and therapeutic effects analysis of potential bioactive compounds group (BCG) of CB. **A** Heatmap of the potential interventional effects of compounds contained in CB on four syndromes. **B** Differences between potential BCG and the whole formula of CB’s intervention in leukopenia. **C** Multi-level intervention network of potential BCG to four syndromes of leukopenia. **D** Significant pathways of potential BCG in leukocytes and lymphocyte

Focusing on the TCM syndromes of leukopenia, we screened and identified five compounds significantly intervening in four TCM symptoms, including FD [32], WSK [65], IB and CDFS [2]. Based on the established rules for evaluating the intervention of compounds on TCM syndromes, we collected CTD genes associated with these four representative TCM syndromes of leukopenia (see Supplementary File). It was showed that the PeGs-class compounds, including 3-hydroxytyrosol 3-*O*-glucoside and salidroside, present in the ministerial herb NZZ, along with the terpenoids-class compound syringin, found in the sovereign herb CWJ, exhibit potential intervention effects across all four TCM syndromes of leukopenia [11]. Meanwhile, the lignans-class compound schisandrin, contained in WWZ and the flavonoids-class compound kaempferol-3-glucorhamnoside, found in BHSSC, not only serve as quality control markers for these herbal medicines but also significantly intervene in two key TCM syndromes of leukopenia (IB and WSK, Fig. 5A).

Consequently, we identified these five compounds as the bioactive compounds group (BCG) of CB, calculated their collective target profile, and analyzed the differences between the BCG (CB_chemical) and the complete prescription of the capsule (CB_formula). Enrichment analysis revealed seven overlapping pathways shared by CB_chemical and CB_formula, encompassing modules related to immunity, signaling, and cellular processes. These pathways include response to interferon-gamma, activation of immune response, and phagocytosis (Fig. 5B). Moreover, the effects of CB_formula were more significant compared to CB_chemical, which indirectly highlights the synergistic effects of TCM formulas. Furthermore, the BCG was enriched in GO terms associated with leukocytes and lymphocytes, indicating potential intervention effects of the drug combination on cellular processes involving white blood cells and related immune cells (Fig. 5D).

We further input the CTD genes associated with the four TCM syndromes of leukopenia into the STRING database to construct a PPI network and subsequently established a multi-level intervention network linking the drug combinations to the syndromes (Fig. 5C). In this network, connections between the compound-level modules and syndrome-level modules indicate that the compounds within these modules have potential intervention effects on the corresponding TCM syndromes. The representative compounds from the sovereign herbs and ministerial herbs exhibit potential intervention effects across all four syndromes, while the representative compounds from the adjuvant and guiding herbs demonstrate more pronounced effects specifically on the blood deficiency syndrome.

### CB’s bioactive compounds group protected lymphocytes from depletion under irradiation

**Fig. 6:**
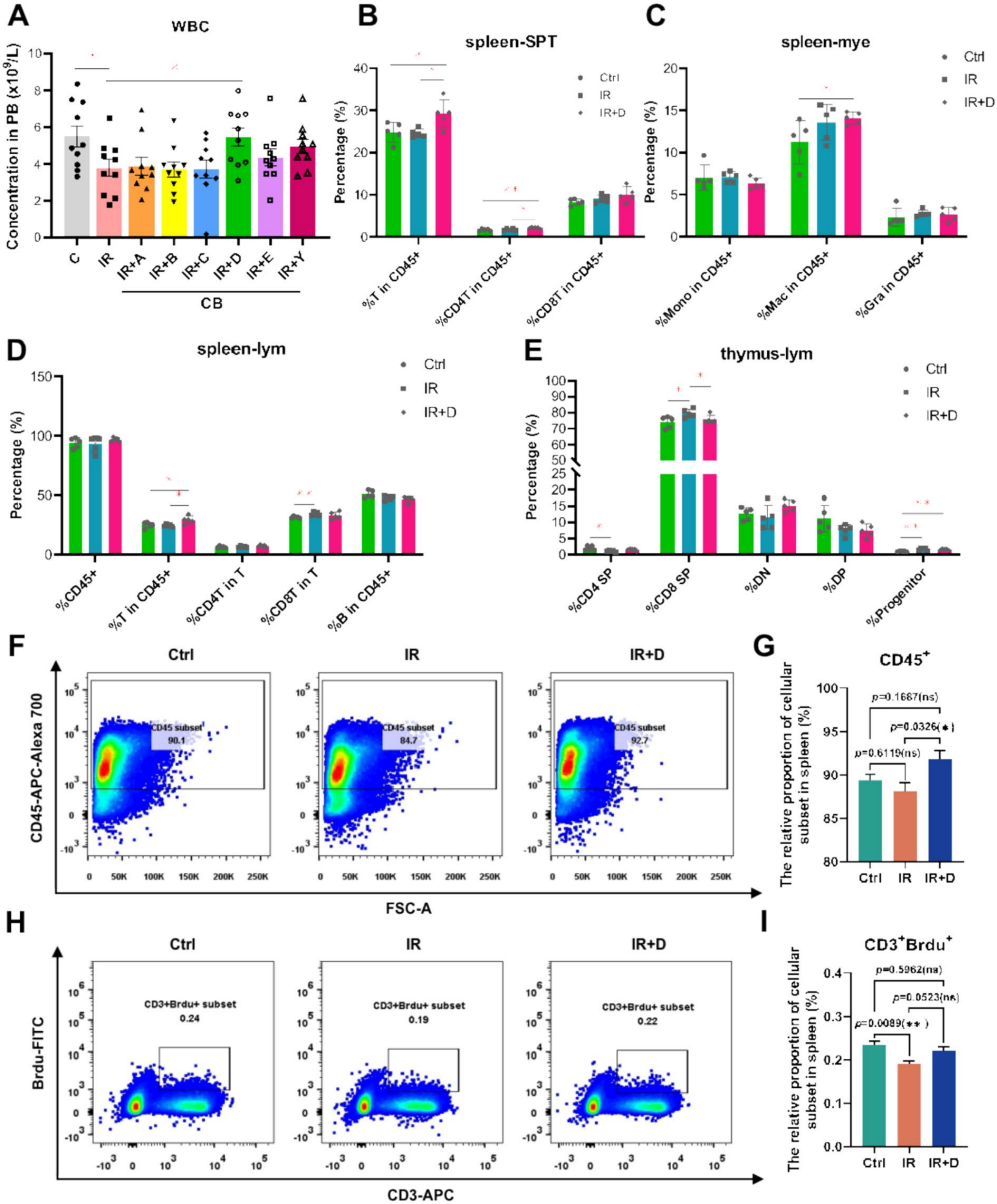
CB exhibits a radioprotective effect on splenic lymphocytes. **A** Number of peripheral blood leukocytes after CB multi-component compatibility treatment. **B**–**D** Percentage of T-cell-related populations in the spleen following drug treatment in group D. **E** Percentage of T-cell-related populations in the thymus following drug treatment in group D. DN, double-negative T cell, DP, double-positive T cell. **F** Representative flow cytometry plots showing the proportion of leukocytes in the spleen across different groups. **G** Statistical graph of leukocyte proportions in the spleen. Data are presented as mean ± SEM. *p < 0.05 (t-test). **H** Representative flow cytometry plots demonstrating changes in T-lymphocyte proliferation in the spleen across different groups. **I** Statistical graph of T-lymphocyte proliferation in the spleen. Data are presented as mean ± SEM. **p < 0.01 (t-test)

To investigate the radioprotective effects of CB multi-component compatibility on immune damage, mice were subjected to 0.1 Gy of ionizing radiation for 5 consecutive days, with drug administration via gavage immediately following each irradiation session. On the sixth day post-irradiation, immune-related parameters were analyzed in eight groups of mice. These groups included non-irradiated controls (Ctrl), irradiated mice administered normal saline via gavage (IR), and irradiated mice treated with CB multi-component compatibility via gavage (IR + A, B, C or D, Table 1). Each group comprised ten mice. Continuous exposure to radiation resulted in a significant decrease in peripheral blood leukocyte counts in the IR group, affecting lymphocytes, myeloid cells, and megakaryocytes. Notably, treatment with group D demonstrated a protective effect on lymphocyte populations (Fig. 6A).

The percentage of T cells among nucleated cells in the spleen exceeds 40%, prompting us to focus specifically on the T cell population. Additionally, we conducted a lineage-level analysis of the thymus to determine whether the T cells present in the spleen originated from this site. Our analysis revealed that group D significantly increased the proportion of T cells within white blood cells in the spleen (Fig. 6B–D). Given that the thymus serves as the primary organ for T-cell differentiation, development, and maturation [62], we also tested the variations in T-cell subsets within the thymus across different groups. In the thymus, compared with the IR group, the proportion of progenitor cells was higher in group D than in the drug treatment group; however, the proportion of single positive T cells did not increase (Fig. 6E). We assessed leukocyte abundance and T-lymphocyte proliferation in the spleen via flow cytometry and found the proportion of CD45⁺ leukocytes in the spleen was significantly increased in group D compared with the IR group (p < 0.05), suggesting a general enhancement of immune cell recovery (Fig. 6F, G). Furthermore, analysis of T-cell proliferation using Ki-67 staining revealed that the proportion of proliferating T lymphocytes (CD3⁺Ki-67⁺) was markedly elevated in group D relative to both the IR and drug-only treatment groups (Fig. 6H–I). This suggests that the number of T cells migrating from the thymus to the spleen would not be augmented. The observed increase in the proportion of T cells in the spleen may instead be attributed to enhanced proliferation of naïve T cells. These findings indicate that the protective effect of group D on T cells likely occurs in situ within the spleen.

### In-depth RNA-seq analysis and quantitative target validation uncovers potential mechanism underlying the intervention of CB’s bioactive compounds group in leukopenia

**Fig. 7:**
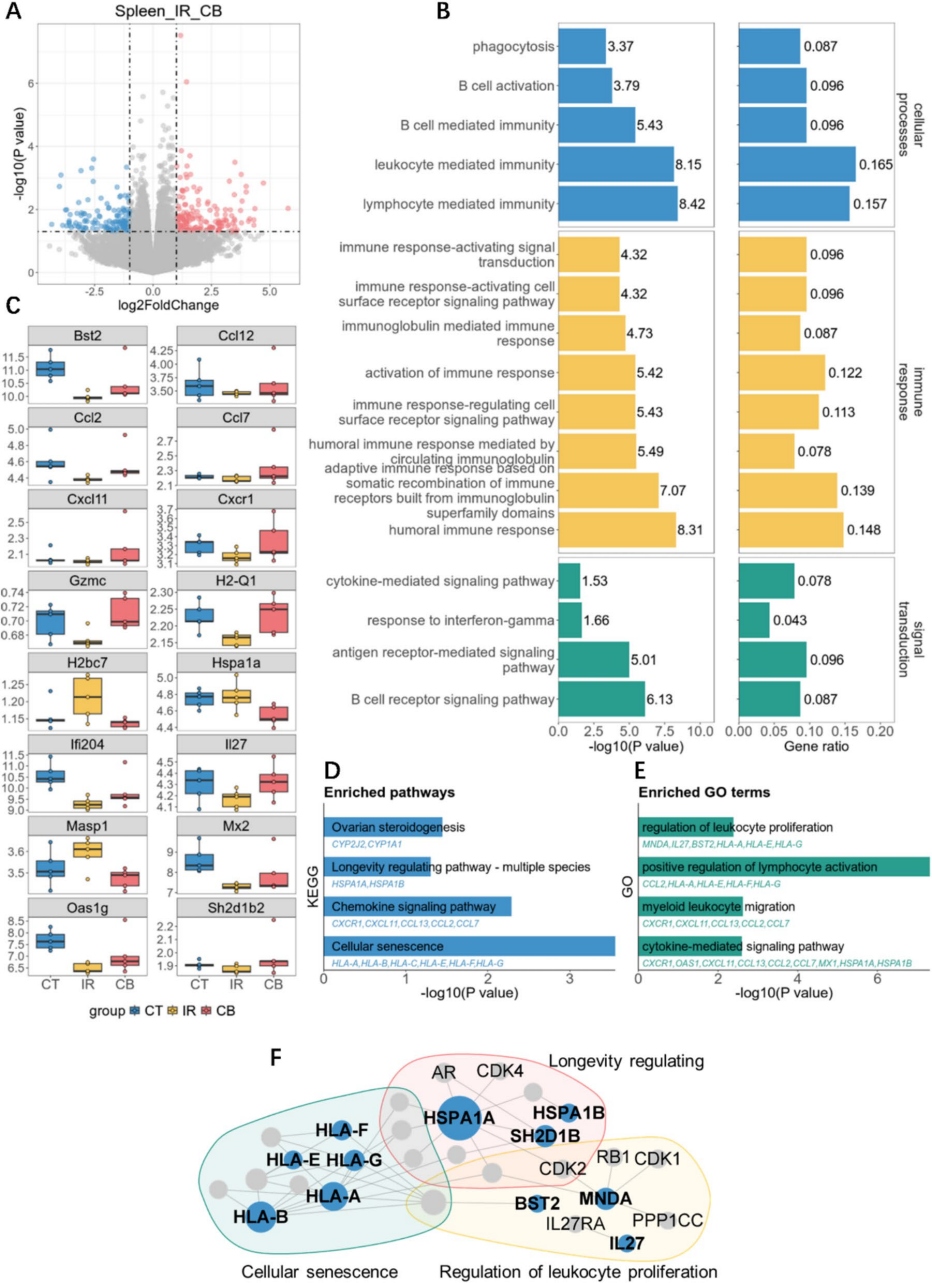
RNA-seq analysis of CB’s bioactive compounds group (BCG) intervention in leukopenia. **A** Volcano plots for differential expressed genes before and after the intervention of potential BCG of CB. **B** Enrichment result of the significantly changed biological processes before and after the intervention of potential BCG of CB. **C** The expression of 16 genes significantly changed before and after the intervention of potential BCG of CB. **D**, **E** Enriched pathways and biological terms of significantly changed genes. **F** Biological network between significantly changed genes before and after the intervention of potential BCG of CB and network targets of potential BCG of CB

To investigate the mechanisms underlying the radioprotective effects of the bioactive compounds in CB, we analyzed gene expression profiles in the spleens of mice from the control group (CT), the irradiated group (IR), and the irradiated group treated with CB’s BCG. Analyzing the transcriptomic differences between the CB group and IR group, we observed that 161 genes were upregulated and 132 genes downregulated (Fig. 7A). The differential gene enrichment analysis confirmed significant effects on 17 of the 22 pathways involved in the full formula intervention of leukopenia (Fig. 7B). These pathways impact cellular processes in leukocytes such as lymphocytes and B cells, regulate leukocyte counts, and influence immune-response activating signal transduction pathways such as the B cell signaling pathway, thus enhancing the body’s immune function and subsequently elevating leukocyte levels.

Analysis of the differential genes in the CT vs IR and IR vs CB groups revealed that CB’s BCG intervention reversed the expression of 16 genes from the model group changes (Fig. 7C), and these genes, along with the predicted targets of CB’s BCG, were part of the same biological network (Fig. 7F). Central to this network, besides immune response cytokines like HLA-B and HLA-A, HSPAIA, MNDA and BST2 are also key roles regulating this network. MNDA is a nuclear protein predominantly expressed in myeloid cells like monocytes and neutrophils [7, 45], involved in immune response and DNA-binding signal transduction [20, 46, 48]. HSPA1A is a heat shock protein involving in protein folding, cell protection, and stress responses [67], regulating apoptosis, cell proliferation, and immune signal transduction. By protecting cells from oxidative stress or cytotoxicity, HSPA1A plays a significant role in maintaining homeostasis and supporting white blood cell proliferation, especially under stress conditions.

Building on insights from flow cytometric analysis, we conducted KEGG pathway enrichment analysis of DEGs and identified four pathways that were simultaneously enriched in both the overall target spectrum of the compound combination and the DEGs, including ovarian steroidogenesis, longevity regulating pathway, chemokine signaling pathway, and cellular senescence (Fig. 7D). Furthermore, GO enrichment analysis revealed four significantly enriched terms related to leukocyte proliferation, differentiation, and activation: regulation of leukocyte proliferation, positive regulation of lymphocyte activation, myeloid leukocyte migration, and cytokine-mediated signaling pathway (Fig. 7E). Notably, key genes such as MNDA, HSPA1A, HLA-A, HLA-G, and BST2, which are central and densely connected within the biological network, were also involved in these pathways (Fig. 7F). These findings suggest that CB’s BCG exerts its effects through multiple pathways and gene networks, facilitating leukocyte proliferation and immune recovery.

**Fig. 8:**
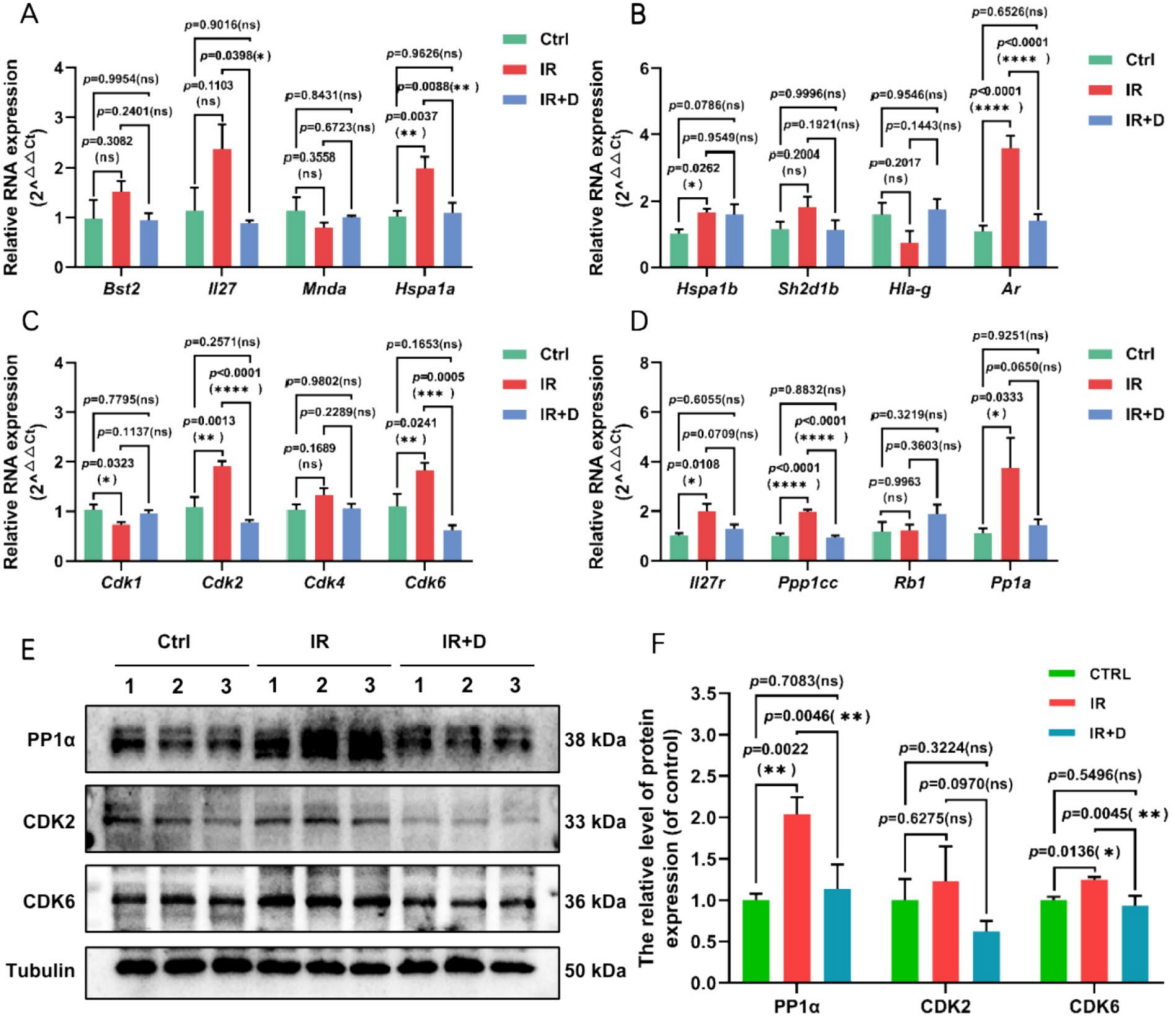
Validation of core target genes and proteins of CB’s bioactive compounds group (BCG) intervention in leukopenia. **A**–**D** The relative expression of Bst2, Il27, Mnda, Hspa1a, Hspa1b, Sh2d1b, Hla-g, Ar, Cdk1, Cdk2, Cdk4, Cdk6, Il27ra, Ppp1cc, Rb1, Pp1a in Spleen from indicated groups. Gapdh was used as reference gene for relative quantification of RNA expression (n = 3). Data are presented as mean ± SEM.*p < 0.05, **p < 0.01,***p < 0.001, ****p < 0.0001 (t-test). **E**, **F** Western blotting analysis of PP1α, CDK2, CDK6 protein expression in Spleen of mice. Data are presented as mean ± SEM.*p < 0.05, **p < 0.01 (t-test).

To further validate the transcriptomic predictions, we performed reverse transcription quantitative polymerase chain reaction (RT-qPCR) and Western blot (WB) assays on selected core targets. Genes including PPP1CA, RB, CDK1/2/4/6, MNDA, BST2, and HSPA1A were selected based on their centrality and biological relevance within the network. We found RT-qPCR resulting a significance regulation of core genes like HSPA1A, AR, CDK2, CDK6 and PPP1CC, all of which are critical regulators of cell cycle progression (Fig. 8A–D). Further WB results also demonstrated a significant upregulation of CDK2, CDK6, and PPP1CC (Fig. 8E, F). Notably, CDK2 and CDK6, two core regulators of G1-S phase transition suggesting a potential enhancement of hematopoietic stem/progenitor cell proliferation. Similarly, PPP1CC, a catalytic subunit of protein phosphatase 1 involved in cell cycle control and DNA damage repair, was also markedly upregulated. These molecular validations support the hypothesis that CB promotes hematopoietic recovery by modulating cell cycle-related pathways, alleviating DNA damage, and enhancing hematopoietic stem cell regeneration following radiation-induced injury.

## Discussion

Ionizing radiation carries a significant risk of causing leukemia. It has been shown that there is a positive dose–response relationship between long-term exposure to low-dose radiation environments and subsequent death due to leukemia [36]. It is critical to develop therapeutic agents that are effective against leukopenia in exposing to low-dose radiation. However, current commonly used therapeutic options, such as granulocyte macrophage-colony stimulating factor (GM-CSF) and granulocyte-colony stimulating factor (G-CSF), are limited and often ineffective, with significant adverse effects [10, 17].

In clinical settings, the patients commonly manifests with symptoms such as fatigue, dizziness, palpitations, insomnia, poor appetite, low-grade fever, sore throat, and tongue ulcers [22]. These symptoms, when analyzed through the TCM framework, align with the syndromes of blood deficiency, fatigue syndrome and warm disease. CB, which is known to nourish the kidney [29] and spleen [8] while enriching and replenishing the blood, has effects that match to these symptom patterns. Studies have demonstrated that CB effectively promote the recovery of peripheral blood counts and hematopoietic colonies after low-dose radiation exposure, significantly increasing WBC levels [26].

Through network pharmacology analysis combined with transcriptomic studies, we identified that CB potentially intervene in leukopenia through 22 pathways related to cellular process, immune response, and signal transduction. WBCs are classified as lymphocytes, basophils, neutrophils, eosinophils and monocytes [15]. It has been proposed that there is a correlation between leukopenia and changes in the immune milieu, with WBC counts being affected when changes in regulatory T-cell values and IL-7 are produced [14]. Further analysis of the impact of CB components on TCM syndromes such as blood deficiency, fatigue syndrome, warm disease, and spleen-kidney weakness led to the identification of five key compounds as a combination with a significant effect on syndrome modulation. These compounds include kaempferol-3-glucorhamnoside, syringin, schisandrin, 3-hydroxytyrosol-3-*O*-glucoside and salidroside are all quality controlled compounds of herbs contained in CB. Within them, syringin [12, 57, 68, 76] and schisandrin [21, 50, 72] have been found by researchers with medical value in terms of antioxidants and regulating the body’s immunity. Further network target analysis also indicated that this combination notably influences immune signaling pathways and leukocyte- and lymphocyte-related cellular processes.

We determined the effective dosage of this compound combination and validated its efficacy through flow cytometric analysis, confirming its role in increasing peripheral WBC counts and promoting splenic immune cell proliferation in mice. Additionally, RNA-seq analysis revealed that these five key compounds may exert their effects by targeting PP1A, RB, CDK4/6, CDK2, and CDK1, thereby indirectly modulating key molecules such as MNDA, BST2, and HSPA1A. HSPA1A, BST2 and MNDA have high degree in the PPI network, moreover are central genes in functions of controlling the leukocyte cell cycle and affecting its proliferation apoptosis and immunoregulation. These molecules are involved in immune regulation and cellular proliferation, supporting the mitigation of radiation-induced leukopenia through immune and hematopoietic recovery.

Furthermore, we established the association of compounds of CB and potential targets affecting leukopenia by network target analysis [33]. The positioning of BCG was achieved by combination of TCM syndromes and further verified by ex vivo and in vivo experiments. The result showed that CB’s BCG can effectively inhibit radiation-induced lymphocytopenia. We also analyzed the potential intervention mechanisms with RNA-seq results and proposed core targets such as PP1A, RB, MNDA, BST2, and HSPA1A.

**Fig. 9:**
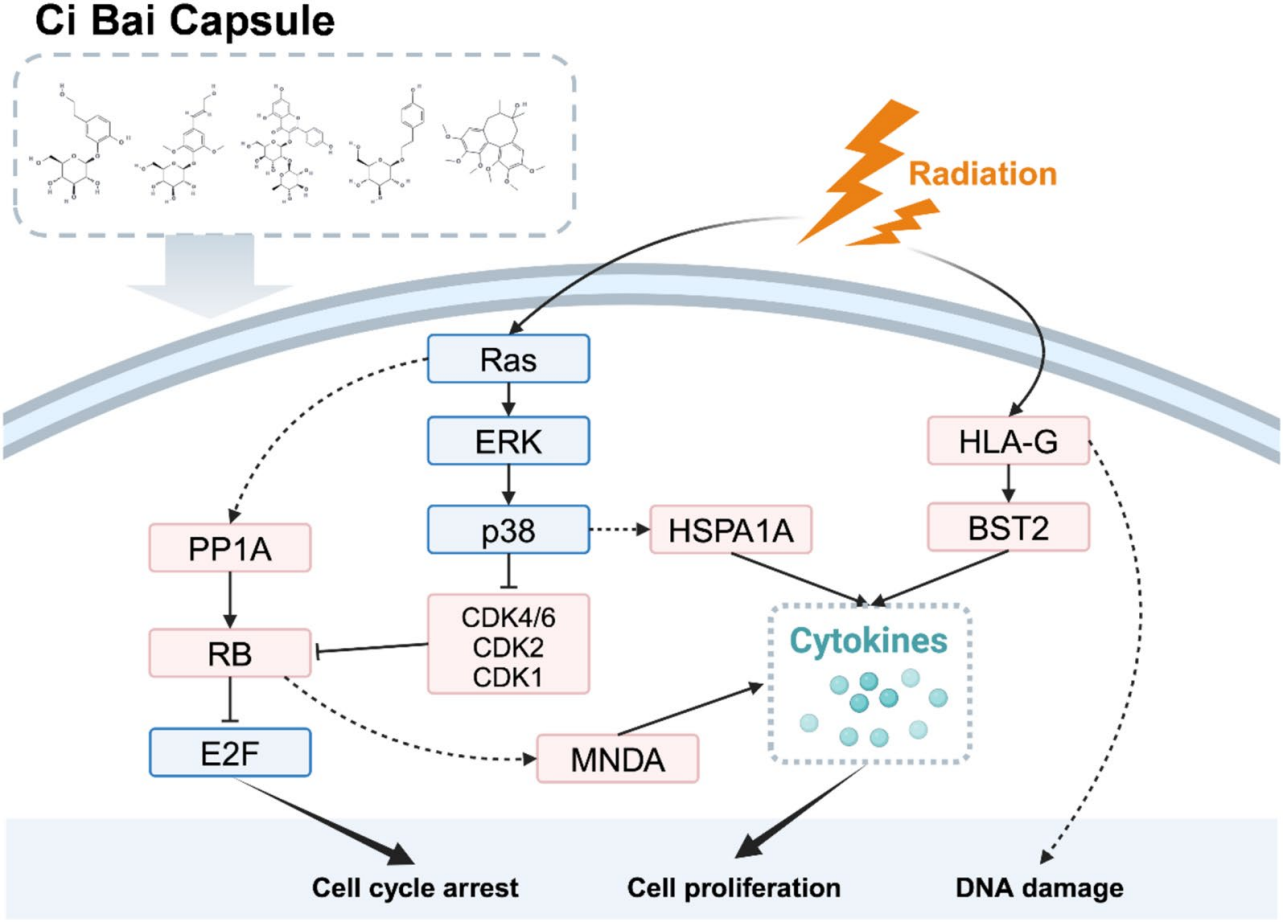
Mechanistic pathways of CB’s potential bioactive compounds group (BCG) in mitigating radiation-induced leukopenia. Radiation initiates DNA damage via ATM/ATR-p53 activation and ROS overproduction, triggering p38 MAPK-mediated HSPA1A upregulation for stress adaptation and apoptosis resistance. Concurrently, MNDA promoters NF-κB-driven myelopoiesis while BST2 enhances antiviral immunity through myeloid proliferation. CB’s BCG synergistically sustains leukocyte homeostasis by coupling oxidative defense (HSPA1A), differentiation coordination (MNDA), and immune optimization (BST2). CB’s BCG amplify these effects by modulating cell cycle regulators (PP1A/RB/CDKs), resolving DNA damage and promoting cell proliferation

Based on a literature review and the gene relationships within cellular senescence, we inferred the regulatory mechanisms by which the potential BCG of CB alleviates leukopenia (Fig. 9). Radiation exposure triggers DNA damage through the ATM/ATR-p53 pathways and induces reactive oxygen species (ROS) accumulation [13, 38], activating the p38 MAPK pathway to upregulate HSPA1A expression for stress resistance and anti-apoptosis [61]. Simultaneously, MNDA enhance myelopoiesis via NF-κB signaling and inflammatory cytokine regulation, and BST2 reinforces innate immunity by restricting viral particles and myeloid cell proliferation [25]. CB’s BCG synergistically maintains leukocyte homeostasis: HSPA1A preserves cellular homeostasis under oxidative stress, MNDA coordinates differentiation, and BST2 optimizes immune surveillance. Downstream of these targets regulated by CB’s BCG, PP1A, RB, CDK4/6, CDK2, and CDK1 are indirectly modulated, leading to influences of cell cycle, promotion of cell proliferation, and resistance to DNA damage. This multi-target defense network regulated by CB integrates immune response and cellular proliferation to combat radiation-induced leukopenia, highlighting the potential of CB in mitigating immune and hematopoietic damage caused by radiation exposure.

Despite the progress made in this study, there are some limitations that should be acknowledged. First, the indication of BCG did not take dosage into account, which was addressed in follow-up experiments to refine the conclusions. Second, experimental studies are needed to confirm the proposed crosstalk at a functional level.

## Conclusion

This study systematically deciphers the therapeutic mechanism of CB against radiation-induced leukopenia through integrative network pharmacology and experiment validation. From the perspective of the JUN-CHEN-ZUO-SHI classification of TCM, we analyzed the potential mechanisms of the herbs in CB that confer resistance to radiation-induced leukopenia. Through further screening, we identified a BCG comprising five compounds derived from CB. The BCG within CB, dominated by flavonoids and terpenoids, orchestrates HSPA1A, MNDA and BST2 regulatory pathways to counteract radiation damage by modulating cell cycle regulators, resolving DNA damage, and promoting hematopoietic stem cell regeneration. By bridging TCM syndrome patterns with molecular networks, this work establishes a “component-pathway-syndrome” evaluation framework, advancing herbal formula research from empirical application to mechanism-defined therapeutics. These findings not only validate CB’s radioprotective efficacy but also provide a paradigm for modernizing complex traditional formulations through systems biology approaches.

## Supplementary Information

Supplementary Material 1.

## Data Availability

Raw data is available from the corresponding author upon request. All authors agree to be accountable for all aspects of work ensuring integrity and accuracy.

## Abbreviations

CB: Ci Bai Capsule
TCM: Traditional Chinese Medicine
WBC: White blood cell
BHSSC: *Scleromitrion diffusum*
CWJ: *Eleutherococcus senticosus*
FL: *Poria*
BZ: *Atractylodes macrocephala* Koidz
DS: *Codonopsis radix*
HZ: *Reynoutria japonica*
GQZ: *Lycium chinense*
TSZ: *Cuscuta*
WWZ: *Schisandra chinensis*
NZZ: *Ligustri lucidi* Fructus
FD: Febrile disease
WSK: Weakened spleen and kidneys
IB: Insufficient blood
CDFS: Chronic Deficiency or Fatigue Syndrome
JUN: Sovereign
CHEN: Minister
ZUO/SHI: Assistant
