# Supplementary material for: Exploring the potential bioactive compounds group and mechanism of Ci Bai Capsule in treating leukopenia: a combined approach of network pharmacology and transcriptome evidences
Source: Chin Med. 2025 Sep 1;20:139. doi: 10.1186/s13020-025-01197-9 (PMC12400580; doi:10.1186/s13020-025-01197-9)
Supplement: Supplementary file 1 — Supplementary Material 1. [file 13020_2025_1197_MOESM1_ESM.zip › Supplementary file/suppl3-Collection of components in Cibai Capsule.docx]

| Collection of components in Cibai Capsule | | | |
| --- | --- | --- | --- |
| **Herb** | **Compound classification** | **Compound name** | **Ref.** |
| **CWJ** | Terpenoids | syringin, oleanolic acid, ursolic acid, etc. | [1-5] |
|  | Lignans | eleutheroside E, etc. |  |
|  | Flavonoids | hyperoside, quercetin, rutin, etc. |  |
|  | Coumarins | isozinpyridine, etc. |  |
| **BHSSC** | Terpenoids | Cyclen ether terpenes, triterpenes (oleanolic acid, ursolic acid, bicoumaric acid, etc.) | [6-9] |
|  | Flavonoids | Kaempferol, quercetin, rutin, etc. |  |
|  | Sterols | β-Sitosterol, Soy Sterols, Carotenoids, etc. |  |
|  | Polysaccharides | Heteropolysaccharides composed mainly of rhamnose, glucose, galactose, arabinose and mannose |  |
| **WWZ** | Lignans | Schisandrin A, Schisandrin B, Schisandrin A, Schisandrin B, Schisandrin A, Schisandrin B, etc. | [10-14] |
|  | polysaccharides | Polysaccharide-B Ⅰ/Ⅱ/Ⅲ and other polysaccharide fractions of Fructus schisandrae chinensis |  |
|  | Flavonoids | Quercetin, apigenin, populin, kaempferol, lignocerotoxin, etc. |  |
|  | Terpenoids | Betulinic acid, glycolic acid, oleanolic acid, etc. |  |
| **NZZ** | Terpenoids | Oleanolic acid, ursolic acid, Teicoplanin, oleoside dimethyl ester, olive bitter glycoside, neo-ligustroside, and others. | [15,16] |
|  | PeGs | Rhodiola rosea glycosides, mullein, pinacoside, norbornanin and tyrosol. |  |
|  | Flavonoids | Apigenin, lignans, quercetin, Douglas fir, and lignans-7-O-β-D-glucoside. |  |
|  | Others | Polysaccharides, fatty acids, amino acids and trace elements, etc. |  |
| **TSZ** | Flavonoids | Hypericin, echinacoside, isoquercitrin, isorhamnetin, kaempferol, quercetin and its derived glycosides, etc. | [17,18] |
|  | Phenolic acid | Chlorogenic acid, isochlorogenic acid, caffeic acid, p-coumaric acid, cryptochlorogenic acid, cinnamic acid, etc. |  |
|  | Polysaccharide | A heteropolysaccharide composed of glucose, galactose, rhamnose, mannose, and glucuronic acid. |  |
|  | Lignans | Cuscutaoside A, Cuscutaoside D, D-pinoresinol-4-O-glucopyranose, neocuscutaoside C, etc. |  |
| **HZ** | Flavonoids | β-sitosterol, carotenoids, apigenin, hesperidin, rutin, quercetin, genistein, etc. | [19,20] |
|  | Anthraquinone | Rheumatoid, Rheumatoid acid, Rheumatol, Rheumatoxin methyl ether, etc. |  |
|  | Coumarins | 7-Hydroxy-4-methoxy-5-methylcoumarin |  |
|  | Others | Organic acids and some trace elements |  |
| **DS** | Polyacetylene | Diosgenin A-G, Diosgenin, Diosgeninin, Diosgeninin, Diosgeninol, etc. | [21-23] |
|  | Flavonoids | Baicalein, quercetin, lignan, kaempferol, lignanoside, etc. |  |
|  | Lignans | Eugenol, Arachidonic acid A, Codonopsis pilosulae I-VI, etc. |  |
|  | Terpenoids | Atractylenolide Ⅰ, cork ketone, lupin, oleanolic acid, etc. |  |
|  | Steroid | Mainly porstanol and leguminous glycosides, etc. |  |
|  | Sugar | Polysaccharides (consisting of inulin-type fructans and other heteropolysaccharides) and free sugars (glucose, sucrose, fructose, D-mannose, etc.) |  |
|  | Others | Organic acids, volatile oils, amino acids and trace elements, etc. |  |
| **GQZ** | polysaccharide | LBP (glucose, rhamnose, galactose, arabinose, mannose, fucose, xylose, etc.) | [24-27] |
|  | Flavonoids | Rutin, quercetin, kaempferol, chrysin, mulberry pigment, populin, etc. |  |
|  | Coumarins | Scopoletin, isoscopoletin, heptaphyllum lactone, etc. |  |
|  | Pigment | Composed of carotenoids (free carotenoids and carotenoid fatty acid esters) and lutein-like colored substances, collectively known as wolfberry pigments |  |
|  | Amino acids and vitamins | Contains approximately 19 amino acids (arginine, proline, leucine, isoleucine, valine, etc.), and is currently the only phytosome reported to contain taurine components |  |
|  | Trace elements and inorganic salts | Large amounts of trace elements including iron, copper, zinc, germanium, manganese, magnesium, calcium, potassium, copper, zinc, iron, etc. |  |
|  | Others | Pyrrole derivatives and superoxide dismutase, etc. |  |
| **BZ** | Volatile oil | Atractylenolide Ⅰ～Ⅶ, Atractylenolone, Atractylenolactam, etc. | [28,29] |
|  | Polysaccharide | Mainly atractylodesmus polysaccharides (YY13008, PAM, WAM, PAMS1, PAMS2, AMP, etc.) |  |
|  | Organic acid | Isochlorogenic acid B, isochlorogenic acid A, isochlorogenic acid C, etc. |  |
|  | Others | Essential amino acids |  |
| **FL** | Polysaccharides | Pachytene glycosides as the main component | [28, 30-31] |
|  | Terpenoids | 4 classical triterpenoids of lanoster-8-en-type triterpenes, lanoster-7,9(11)-dien-type triterpenes, 3,4-open-cyclo-8-en-type triterpenes and 3,4-open-cyclo-linoster-7,9(11)-dien-type triterpenes, as well as other triterpenoids and steroids (oleanolic acid, oleanolic acid, lupinol, etc.) |  |
|  | Others | Some proteins, amino acids and trace elements |  |

**References:**

1. Li T, Ferns K, Yan Z Q, et al. Acanthopanax senticosus: Photochemistry and anticancerpotential [J]. Am J Chin Med, 2016, 44(8): 1543-1558.
2. HAN Minghu, HU Haobin, LU Yani, et al. Progress in the study of chemical constituents of Wuga [J]. Chinese Materia Medica, 2019, 42(11): 2720-2729.
3. Liu Y, Wang Z, Wang C, et al. Comprehensive phytochemical analysis and sedative-hypnotic activity of two Acanthopanax species leaves. Food Funct. 2021 Food Funct. 2021 Mar 15;12(5):2292-2311.
4. K.-X. Wu, J. Liu, Y. Liu, X.-R. Guo, L.-Q. Mu, X.-H. Hu and Z.-H. Tang, A comparative metabolomics analysis reveals the tissue-specific phenolic profiling in two acanthopanax species, Molecules, 2018, 23, 2078.
5. Y.-H. Wang, Y. Meng, C. Zhai, M. Wang, B. Avula, J. Yuk,K. M. Smith, G. Isaac and I. A. Khan, The Chemical Characterization of Eleutherococcus senticosus and Ci-wu-jia Tea using UHPLC-UV-QTOF/MS, Int. J. Mol. Sci., 2019,20, 475.
6. Li N, Liu C, Mi S, et al. Simultaneous determination of oleanolic acid, p-coumaric acid, ferulic acid, kaemperol and quercetin in rat plasma by LC-MS-MS and application to a pharmacokinetic study of Oldenlandia diffusa extract in rats. J Chromatogr Sci. 2012 Nov-Dec;50(10):885-92.
7. Ganbold M, Barker J, Ma R, et al. Cytotoxicity and bioavailability studies on a decoction of Oldenlandia diffusa and its fractions separated by HPLC. j Ethnopharmacol. 2010 Sep 15;131(2):396-403.
8. Ganbold M, Barker J, Ma R, et al. Cytotoxicity and bioavailability studies on a decoction of Oldenlandia diffusa and its fractions separated by HPLC. j Ethnopharmacol. 2010 Sep 15;131(2):396-403.
9. Liang Z, Jiang Z, Ho H, et al. Comparative analysis of Oldenlandia diffusa and its substitutes by high performance liquid chromatographic fingerprint Comparative analysis of Oldenlandia diffusa and its substitutes by high performance liquid chromatographic fingerprint and mass spectrometric analysis. Planta Med. 2007 Nov;73(14):1502-8.
10. Wang J, Jiang B, Shan Y, et al. Metabolic mapping of Schisandra chinensis lignans and their metabolites in rats using a metabolomic approach based on HPLC with quadrupole time-of-flight MS/MS spectrometry. J Sep Sci. 2020 Jan;43(2):378-388.
11. Jiang P, Lu Y, Chen D. Authentication of Schisandra chinensis and Schisandra sphenanthera in Chinese patent medicines. J Pharm Biomed Anal. 2016 Nov 30;. 131:263-271.
12. Bao S-y, Han S-y, Cheli-geer C-r and W-l A, Effects of agiophyllumoligo saccharides on insulin resistance of Goto-Kakizaki rats. chin Pharmcol Bull 32. 403-409 (2016).
13. Wei B, Li Q, Su D, et al. Development of a UFLC-MS/MS method for simultaneous determination of six lignans of Schisandra chinensis (Turcz.) Baill. in rat plasma and its application to a comparative pharmacokinetic study in normal and insomnic rats. J Pharm Biomed Anal. 2013 Apr 15;77:120-7.
14. Gao S, Chen H, Zhou X. Study on the spectrum-effect relationship of the xanthine oxidase inhibitory activity of Ligustrum lucidum. j Sep Sci. 2019 Nov;42( 21):3281-3292.
15. Wang Y, Feng K, Li M, et al. Identification of prototypes from Ligustri Lucidi Fructus in rat plasma based on a data-dependent acquisition and multicomponent pharmacokinetic study. Biomed Chromatogr. 2020 Jul;34(7):e4833.
16. Zhang D, Sun L, Li H, et al. Pharmacokinetic comparison of nine bioactive components in rat plasma following oral administration of raw and wine- processed Ligustri Lucidi Fructus by ultra-high-performance liquid chromatography coupled with triple quadrupole mass spectrometry. J Sep Sci. 2020 Nov;43(21):3995-4005.
17. He X, Yang W, Ye M, et al. Differentiation of Cuscuta chinensis and Cuscuta australis by HPLC-DAD-MS analysis and HPLC-UV quantitation. Planta Med. 2011 Nov;77(17):1950-7.
18. Wang H, Hou X, Li B, et al. Study on Active Components of Cuscuta chinensis Promoting Neural Stem Cells Proliferation: Bioassay-Guided Fractionation. Molecules. 2021 Nov 2;26(21):6634.
19. SUN Yinshi,WANG Jianhua. Study on the chemical constituents of Rhizoma Pinelliae[J]. Chinese Herbal Medicine,2015,46(15):2219-2222.
20. Wang HL, Gao JP, Han YL, et al. Comparative studies of polydatin and resveratrol on mutual transformation and antioxidative effect in vivo. Phytomedicine. 2015 May 15;22(5):553-9.
21. Kim EY, Kim JA, Jeon HJ, et al. Chemical fingerprinting of Codonopsis pilosula and simultaneous analysis of its major components by HPLC-UV. Arch Pharm. Res. 2014;37(9):1148-58.
22. Liu W, Lv X, Huang W, et al. Characterization and hypoglycemic effect of a neutral polysaccharide extracted from the residue of Codonopsis Pilosula. Carbohydr Polym. 2018 Oct 1;197:215-226.
23. Zeng X, Li J, Lyu X, et al. Untargeted Metabolomics Reveals Multiple Phytometabolites in the Agricultural Waste Materials and Medicinal Materials of Codonopsis pilosula. Front Plant Sci. 2022 Jan 10;12:814011.
24. ZHANG Qili, CUI Zhijia, YANG Maomao, XIA Pengfei, LI Fuyun, JIN Ling, MA Yi, WANG Zhenheng, ZHAO Wenlong. Differential evaluation of Lycium barbarum from different origins based on HPLC fingerprinting[J]. International Journal of Pharmaceutical Research,2019,46(03):232-237.
25. LUO Qing, MI Jia, RAN Linwu, LU Lu, YAN Yamei, LI Xiaoying, LI Yuekun, CAO Youlong, ZHOU Xuan. Research progress of carotenoids in Lycium barbarum[J]. Food Industry Science and Technology,2018,39(24):331-335+339.
26. Patsilinakos A, Ragno R, Carradori S, et al. Carotenoid content of Goji berries: CIELAB, HPLC-DAD analyses and quantitative correlation. food Chem. 2018 Dec 1;268:49-56.
27. Li YY, Di R, Hsu WL, et al. Quality control of Lycium chinense and Lycium barbarum cortex (Digupi) by HPLC using kukoamines as markers. chin Med. 2017 Jan 9;. 12:4.
28. LU Guangying, XING Xunyan, WANG Jiayun, WANG Yuan, MA Ke, WANG Shijun. Research progress and predictive analysis of quality markers of the classic formula Ginseng Ling Bai Zhu San[J/OL]. Chinese Journal of Traditional Chinese Medicine:1-13[2022-09-18].
29. Shan GS, Zhang LX, Zhao QM, et al. Metabolomic study of raw and processed Atractylodes macrocephala Koidz by LC-MS. J Pharm Biomed Anal. 2014 Sep;98:74-84 .
30. Zhang Y, Wu M, Xi J, et al. Multiple-fingerprint analysis of Poria cocos polysaccharide by HPLC combined with chemometrics methods. j Pharm Biomed Anal. 2021 May 10;198:114012.
31. Wu LF, Wang KF, Mao X, et al. Screening and Analysis of the Potential Bioactive Components of Poria cocos (Schw.) Wolf by HPLC and HPLC-MS(n) with the Aid of Chemometrics. molecules. 2016 Feb 18;21(2):227.
